# The Effect of Liquid Rubber Addition on the Physicochemical Properties, Cytotoxicity, and Ability to Inhibit Biofilm Formation of Dental Composites

**DOI:** 10.3390/ma14071704

**Published:** 2021-03-30

**Authors:** Krzysztof Pałka, Małgorzata Miazga-Karska, Joanna Pawłat, Joanna Kleczewska, Agata Przekora

**Affiliations:** 1 Faculty of Mechanical Engineering, Lublin University of Technology, Nadbystrzycka 36, 20-618 Lublin, Poland; 2 Chair and Department of Biochemistry and Biotechnology, Medical University of Lublin, Lublin, Chodźki 1, 20-093 Lublin, Poland; malgorzata.miazga-karska@umlub.pl; 3 Institute of Electrical Engineering and Electrotechnologies, Lublin University of Technology, Nadbystrzycka 38A, 20-618 Lublin, Poland; j.pawlat@pollub.pl; 4 Arkona Laboratorium Farmakologii Stomatologicznej, Nasutów 99C, 21-025 Niemce, Poland; joanna@arkonadent.com

**Keywords:** resin composite, wettability, biofilm formation, cytotoxicity

## Abstract

The aim of this study was to evaluate the effect of modification with liquid rubber on the adhesion to tooth tissues (enamel, dentin), wettability and ability to inhibit bacterial biofilm formation of resin-based dental composites. Two commercial composites (Flow-Art–flow type with 60% ceramic filler and Boston–packable type with 78% ceramic filler; both from Arkona Laboratorium Farmakologii Stomatologicznej, Nasutów, Poland) were modified by addition of 5% by weight (of resin) of a liquid methacrylate-terminated polybutadiene. Results showed that modification of the flow type composite significantly (*p* < 0.05) increased the shear bond strength values by 17% for enamel and by 33% for dentine. Addition of liquid rubber significantly (*p* < 0.05) reduced also hydrophilicity of the dental materials since the water contact angle was increased from 81–83° to 87–89°. Interestingly, modified packable type material showed improved antibiofilm activity against *Steptococcus mutans* and *Streptococcus sanguinis* (quantitative assay with crystal violet), but also cytotoxicity against eukaryotic cells since cell viability was reduced to 37% as proven in a direct-contact WST-8 test. Introduction of the same modification to the flow type material significantly improved its antibiofilm properties (biofilm reduction by approximately 6% compared to the unmodified material, *p* < 0.05) without cytotoxic effects against human fibroblasts (cell viability near 100%). Thus, modified flow type composite may be considered as a candidate to be used as restorative material since it exhibits both nontoxicity and antibiofilm properties.

## 1. Introduction

The polymer matrix of dental composites is commonly made of methacrylate resin blends. Unfortunately, due to the brittleness of resin-based composites, they show susceptibility to fracture under the occlusal forces [1]. Most studies focus on the toughening of dental composites by optimization of the resin mixture composition or reinforcement modifications [2,3,4]. Introduction of elastomers into a matrix of the dental composites is a promising method for their toughening and improvement of their fracture toughness [5,6,7]. Until recently, rubber modification of resin-based composites (RBC) remained in the realm of the laboratory [6,8]. Our latest research has shown that modification of the composite matrix with an acrylonitrile-free liquid rubber not only increases the fracture toughness [7], but also ensures patient safety due to the use of materials without carcinogenic properties. The durability and clinical success related to the use of dental restorations highly depend on the strength of adhesive system which provides long-lasting bonds to the hard tooth tissues and the restorative composite. The morphological features of the cavity surface, which are the results of the mechanical and chemical processing, are responsible for the bond strength of the adhesive system. These microirregularities are impregnated by appropriate (meth)acrylate or (meth)acrylamide monomers with both hydrophilic and hydrophobic groups present in the adhesive system. Hydrophilic groups improve the wettability and bond strength to dental hard tissues, while the hydrophobic ones allow the interaction and co-polymerization with the restorative composite. Polymerization of the monomers results in various forms of interpenetrating networks [9]. The presence of liquid rubber in the composition of restoration material may potentially affects the strength of its bonding to the adhesive system. 

The cytotoxicity of the resin composites used in dentistry is primarily associated with the release of residual monomers due to incomplete polymerization processes [10]. Over 30 different agents, some of them cytotoxic, have been isolated from cured dental composites, including compounds such as the main monomers, comonomers, various additives and reaction products [11]. Despite an observed increased degree of conversion after modification of the composites with liquid rubber [7], there is still possibility of the release of small amounts of monomers or hydrolytic degradation products. It is clear that consequences other than strong cytotoxicity, e.g., potential carcinogenic effect or inflammation, are also of high importance for the determination of the biological safety of dental materials.

The BisGMA resin, which is the main component of the composites’ matrix, has polar hydroxyl groups [12]. In this study, a non-polar liquid rubber [13] was used for the modification of dental composites. Addition of non-polar component may potentially increase the contact angle of the resultant materials, making them more hydrophobic. Importantly, it is known that surface wettability has great impact on bacterial colonization and subsequent biofilm formation on dental materials [14,15]. Other authors have already demonstrated that addition of some components to the dental materials results in increased bacterial biofilm formation, leading to the enamel demineralization [16,17] and even gingival inflammation [18].

In our previous work, it was proven that addition of the acrylonitrile-free liquid rubber as a toughening agent results in improved mechanical properties of the resin composites [7]. It was hypothesized that such a chemical modification of dental materials may also significantly change their physicochemical properties like adhesive properties and wettability. Considering all the above mentioned important issues, the primary purpose of this work was to determine whether applied modification of resin-based materials may influence their adhesive, surface wettability and bacterial colonization properties. Moreover, cytotoxicity tests were performed on eukaryotic cells to evaluate the potential clinical usefulness of the modified composites.

## 2. Materials and Methods

### 2.1. Fabrication of Dental Composites

Two commercial composites: Flow-Art–flow type (marked as F) and Boston–packable type (marked as B) (Arkona, Nasutów, Poland) were used as starting research materials. The composites are made of the same components but characterized by different ceramic contents. The detailed chemical composition of the matrix and reinforcement are nor disclosed by the manufacturer. Briefly, the production of the composites was as follows: the matrix was made of Bis-GMA, Bis-EMA, UDMA and TEGDMA dimethacrylate resins (Sigma-Aldrich Chemicals, Munich, Germany) and it was modified with the 5 wt.% addition of a liquid methacrylate-terminated polybutadiene Hypro® 2000X168LC VTB (CAS 68649-04-7, CVC Thermoset Specialties, Moorestown, NJ, USA) having vinyl reactive functional groups. The mixture was supplemented in each case with a photoinitiator (camphorquinone), co-initiator (2-dimethylaminoethyl methacrylate, DMAEMA) and an inhibitor (butylated hydroxytoluene, BHT) (all additives were purchased from Sigma-Aldrich Chemicals). The composite reinforcement was a mixture of pyrogenic silica, Ba-Al-B-Si glass and titanium dioxide added in various proportions: 60% by weight and 78% by weight in the case of flow (F) and packable (B) type of composite, respectively. The composition and the manufacturing of the composites modified with liquid rubber were claimed in the Polish patent application no. P.427219 [19]. Briefly, the base resins were premixed with the liquid rubber and then the first batch of the ceramic phase was introduced into the mixer in order to disperse the copolymer and obtain its homogeneous distribution in the whole volume of the matrix. Subsequently, the second batch of fillers (ceramic phase and other additives) was introduced into the mixer and the mixing process was continued using a vacuum to remove air from the composite. All materials were cured for 20 s in a stainless steel using led lamp (1350 mW/cm^2^ intensity). Curing was performed according to ISO 4049:2019 standard [20]. All tested materials used in the research were prepared by Arkona Laboratorium Farmakologii Stomatologicznej (Nasutów, Poland). Modified flow (F) and packable (B) composites were marked as FM and BM, respectively. 

### 2.2. Wettability Determination

Wettability (surface water contact angle–CA) was determined using a Krüss DSA25E goniometer (Krüss Scientific Instruments, Hamburg, Germany) equipped with CCD camera. Specimens for the wettability test were prepared in a form of discs (15 mm in diameter, 1 mm thick). Water contact angle was studied through the sessile drop method (1 µL of ultrapure water droplet was dosed at 0.16 mL/min flow rate) using static contact angle measurements [21]. The experiments were performed at room temperature (22 °C) after 24-hour polymerization period of the composites (index “1” next to the sample symbol). The measurements were repeated for the samples that were additionally incubated for 24 h in pure deionized water (index “2” next to the sample symbol) to assess whether humidified oral cavity environment may influence composites’ wettability. The value of the water contact angle, characteristic to selected surface, was obtained by averaging the mean contact angles (in fifteen measurements performed for 3 independent samples, n = 3). The unpaired *t* test and the Statistica software (TIBCO Software Inc., Palo Alto, CA, USA) were used to determine statistically significant results between unmodified samples and corresponding modified composites (B was compared to BM and F was compared to FM). Samples incubated in deionized water were not compared to untreated ones (without incubation in water).

### 2.3. Assessment of the Shear Bond Strength to the Tooth Tissues

The shear bond strength (SBS) was examined according to ISO 29022:2013 standard [22]. The use of the SBS methodology was applied to assess the impact of the presence of liquid rubber in the composites on their adhesive properties.

Twenty human molars without caries were used in this study after obtaining informed consent and approval of the Bioethics Committee of Medical University of Lublin (Lublin, Poland, KE-0254/339/2016). Each tooth was recovered after testing and used four times to obtain the required number of repetitions. In total, each material was tested ten times (n = 10) using enamel or dentin [23]. At the same time, this approach allowed us to maintain the randomness of using the teeth to make the results more reliable. Teeth were sectioned by a low speed diamond saw in order to reveal appropriate tissues while constantly keeping the teeth moist. Then, teeth tissues were mounted in cold-curing resin in cylindrical polycarbonate holders, so as to expose the facial enamel or dentin. After the specimens were mounted, their surfaces were abraded on silicon carbide abrasive paper (P600 grit size). Immediately after the abrasive treatment of the surface, it was etched with orthophosphoric acid for 30 s, then rinsed with a strong stream of water and dried. Then the bonding agent was applied (Masterbond, Arkona) and cured for 10 s using a led curing lamp of intensity 1350 mW/cm^2^. The Masterbond is a single bottle, etch-and-rinse adhesive type, which combines the primer and adhesive action. 

Teflon molds with central cylindrical cavity (2.38 mm in diameter, 2 mm thick) were used for the specimens and filled with the appropriate composite followed by polymerization for 20 s. Specimens were then stored for 24 h at 37 °C in distilled water prior to the testing and were then loaded in a universal testing machine (Autograph AG-X plus, Shimadzu, Kyoto, Japan) at a constant crosshead speed of 0.5 mm/min until fracture. The SBS was then calculated as the stress corresponding to maximum load force divided by the area of the bonded surface. The unpaired *t* test and Statistica software were used to determine statistically significant results between unmodified samples and corresponding modified composites (B was compared to BM and F was compared to FM). Results obtained with dentin were not compared to the results obtained with enamel. 

### 2.4. Antibiofilm Activity

#### 2.4.1. Bacterial Culture

Evaluation of biofilm formation on B, BM, F, FM samples was conducted using a modified Tu et al. method [24]. Bacterial adhesion assays were performed using *Streptococcus mutans PCM 2502* and *Streptococcus sanguinis PCM 2335* strains (the strains were obtained from the Polish Collection of Microorganisms PCM, Institute of Immunology and Experimental Therapy, Polish Academy of Sciences, Wroclaw, Poland) as a model of primary colonizer in biofilm formation on dental materials. In our tests reference bacteria species were used to minimize confounding variables. Initially, bacteria were cultured under anaerobic conditions for 48 h at 37 °C on BHI agar (BioMaxima S.A., Lublin, Poland). Each bacterial strain was diluted in BHI broth (BioMaxima S.A.) to get 1.5 × 10^8^ CFU/mL of bacteria for biofilm assays.

#### 2.4.2. Seeding of the Dental Composites with Bacteria

Eighteen disc samples (three for each experiment) with a diameter of approximately 12 mm and a height of 1 mm were sterilized by immersing the specimens for 10 s in 70% ethanol and drying at room temperature. Dry samples were rinsed twice with 200 µL of phosphate buffered saline (PBS, Pan-Biotech, Aidenbach, Germany) and then placed in the wells of 12-well polystyrene plates. Next, 1000 µL of BHI broth were added to each well. Simultaneously, other plates with composite discs were filled up with 1000 µL of BHI broth with 0.25% sucrose, which is a main sugar responsible for biofilm formation on the dental materials. Sucrose was added to broth to determine the changes in bacterial biofilm formation on the surface of B, BM, F, FM samples dependent on the absence or presence of this sugar in the medium. Finally, an amount of 20 µL of bacterial inoculum (1.5 × 10^8^ CFU/mL) was transferred to each well. In the case of mono-species biofilm tests it was *S. sanguinis* or *S. mutans.* For mixed-species biofilm assays it was 10 µL of *S. sanguinis* (1.5 × 10^8^ CFU/mL) and 10 µL of *S. mutans* (1.5 × 10^8^ CFU/mL) added together. Sterility controls (only BHI or BHI+sucrose) and positive biofilm growth controls formed on the polystyrene surface (BHI or BHI + sucrose with bacterial inoculum) were included in all experiments. Plates were anaerobically incubated at 37 ℃ for 48 h.

#### 2.4.3. Quantitative Biofilm Determination

Biofilm determination was performed according to the procedures described in [25,26]. After 48 h incubation of composite samples with bacterial strains, the medium was discarded and the samples were rinsed twice with 200 µL of fresh medium to leave only bacteria attached to abiotic surfaces. Remaining cells that were attached to the discs, were subsequently stained for 10 min. with CV (1 mL of 0.1% crystal violet) at room temperature, what allowed to visualize biofilm architecture. Discs were transferred to fresh wells, washed twice with 500 µL of sterile water to remove any CV that were not bound to bacteria. Finally, discs with biofilm were individually placed for 15 min. into tubes with 1000 µL of 20% acetic acid to allow the dye to solubilize at room temperature and sonicated (3 min.) to disperse the biofilm. Obtained CV/acetic acid solutions were transferred (200 µL) to a new 96-well plate to measure the optical density (OD at 590 nm) of each sample and additionally positive and negative controls. OD determination (200 µL from 1000 µL of one sample) was repeated five times and the average value was calculated. For each group of materials, a negative control (sterility disc control) consisting of the disc sample immersed in proper broth and positive growth controls (*S. mutans* or *S. sanguinis* or mixed *S. mutans* with *S. sanguinis* with proper broth in wells of polystyrene plates) were included. The OD value obtained for the positive controls was considered as equal to 100% biofilm formation. The results obtained with the experiment were checked for statistically significant differences (*p* < 0.05, n = 3) compared to the positive control of biofilm formation according to the unpaired *t* test (GraphPad Prism 5, Version 5.03 GraphPad Software, Inc., San Diego, CA, USA). Moreover, biofilm formation was compared between unmodified samples and corresponding modified composites (B was compared to BM and F was compared to FM). Results obtained with different bacterial species were not compared to each other. 

#### 2.4.4. Qualitative Biofilm Determination by Confocal Microscopy

The aim of the experiment was to visualize the viability and possible adhesion of bacterial cells to the modified materials (B, BM, F, FM) or control polystyrene surface. For this purpose, a Viability/Cytotoxicity Assay kit for Bacteria Live and Dead Cells (Biotium, Fremont, CA, USA) was used. The kit contains dyes that stain live cells (green fluorescence provided by DMAO dye) and dead bacteria (red fluorescence provided by EthD-III dye and green fluorescence provided by DMAO). Based on conducted staining it was possible to evaluate the structure and viability of the biofilm formed on the abiotic surfaces [27,28]. After 48 h of incubation with bacteria suspension (see Section 2.4.2.), the disc samples were subjected to confocal microscopy observation. Firstly, tested discs were gently rinsed with BHI medium (200 µL) to eliminate planktonic bacteria that were loosely attached to the samples and to leave only the biofilm on the surfaces. Then, samples were placed in the wells and stained using fluorescent dyes’ solution prepared in PBS (Pan-Biotech). The staining procedure was carried out according to the manufacturer instructions. After 15 min of incubation at room temperature, bacterial biofilm on the disc surfaces was visualized using confocal laser scanning microscope (CLSM, Olympus Fluoview equipped with FV1000, Olympus, Tokyo, Japan). The mono-species biofilm positive controls or mixed-species biofilm positive control were grown simultaneously on the bottom of polystyrene wells under the same anaerobic conditions (37 °C for 48 h).

### 2.5. Cytotoxicity Evaluation

#### 2.5.1. Eukaryotic Cell Culture

In vitro cell culture experiments were conducted with the use of normal human fibroblast cell line (BJ, ATCC, CRL-2522^TM^), which is a model cell line commonly applied for cytotoxicity tests on new biomaterials and medical devices. The BJ cells were cultured in EMEM medium (ATCC-LGC Standards, Teddington, UK) with the following supplementation: 10% fetal bovine serum (FBS, Pan-Biotech) and penicillin-streptomycin solution (Sigma-Aldrich Chemicals, Warsaw, Poland). The cells were maintained in an incubator at 37 °C (humidified atmosphere of 95%, 5% CO_2_, 95% air). 

#### 2.5.2. Quantitative Evaluation of Cytotoxicity

Cytotoxicity of the composites was evaluated in direct contact with the eukaryotic cells [29]. Prior to the cell seeding, tested materials were sterilized in the same manner as described in Section 2.4.2. Then the samples were placed in a 24-multiwell plate and presoaked for 20 min. in the complete culture medium at 37 °C. Cell culture-treated glass coverslip in the form of disc (13 mm in diameter) served as a control nontoxic material (negative control of cytotoxicity). 5 × 10^4^ of BJ cells were seeded on top surface of the tested materials in 500 μL of the complete culture medium. After 48-h incubation at 37 °C, viability of BJ cells was assessed by colorimetric WST-8 test (Sigma-Aldrich Chemicals, Poland), which was performed according to the manufacturer protocol. The experiment was carried out in triplicate–3 independent samples of B, BM, F, FM, and control material were tested (n = 3). Cells cultured on the control material were considered to reveal 100% viability. Viability of cells grown on the tested materials was expressed in % and calculated based on the absorbance values obtained with cells grown on the control material and cells cultured on the tested samples. The results obtained with WST-8 assay were checked for statistically significant differences (*p* < 0.05) among tested groups (all groups were compared to each other, including control cells) using one-way ANOVA test followed by Tukey’s multiple comparison test (GraphPad Prism 5, Version 5.03).

#### 2.5.3. Qualitative Evaluation of Cytotoxicity

The experiment was performed in direct contact with tested materials according to protocols described previously [30,31]. BJ fibroblasts were seeded on top surface of the materials in the same manner as described above (Section 2.5.2.). Upon 48-h incubation, cells grown on the top surface of the sample discs as well the cells which flawed down and were cultured on the polystyrene around the tested materials were stained using calcein-AM (green fluorescence of live cells) and propidium iodide (red fluorescence of dead cells). The dyes were the components of Live/Dead Double Staining Kit (Sigma-Aldrich Chemicals, Poland). Stained cells (their viability and morphology) were visualized using CLSM (Olympus Fluoview equipped with FV1000).

## 3. Results and Discussion

### 3.1. Wettability Determination

**Figure 1:**
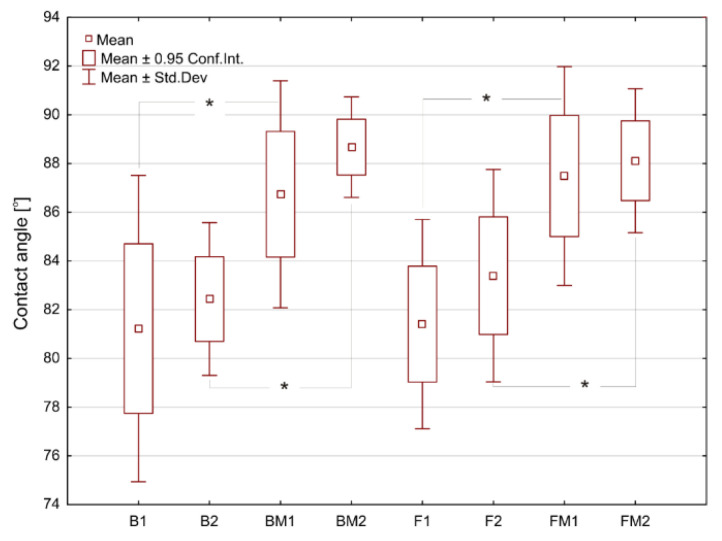
The surface water contact angles determined for tested materials. Asterisks (*) point at statistically significant differences (*p* < 0.05) according to the unpaired *t* test.

Average values of water contact angle (CA) for the tested materials are presented in Figure 1. The pairs of B-F and BM-FM samples had similar water CA values (CA < 90°), indicating their hydrophilic character. Modification of RBC by using liquid rubber significantly (*p* < 0.05) increased the CA value from 81–83° to 87–89°. 

Thus, the introduced modification of dental composites slightly reduced their hydrophilicity. After immersion of the materials in deionized water (for 24 h), a slight increase of water CA values was observed for all samples, however without statistical significance. Wettability of the biomaterials’ surface may have impact on eukaryotic cells’ and bacterium adhesion and thus biocompatibility and biofilm formation on the dental materials, respectively [32,33,34,35]. Results of these studies showed that modification of RBC with liquid rubber reduced their wettability (hydrophilicity). Moreover, humidified oral cavity may further reduce hydrophilicity of fabricated dental composites. The presence of the aquatic environment as well as mechanical interactions, can form–by releasing nonpolar compounds–a thin film on the surface with strong hydrophobic properties. This thin film may provide a strong repulsive force that prevents bacteria adhesion. Similar phenomenon was observed by Rüttermann et al. [36].

### 3.2. Shear Bond Strength

**Figure 2:**
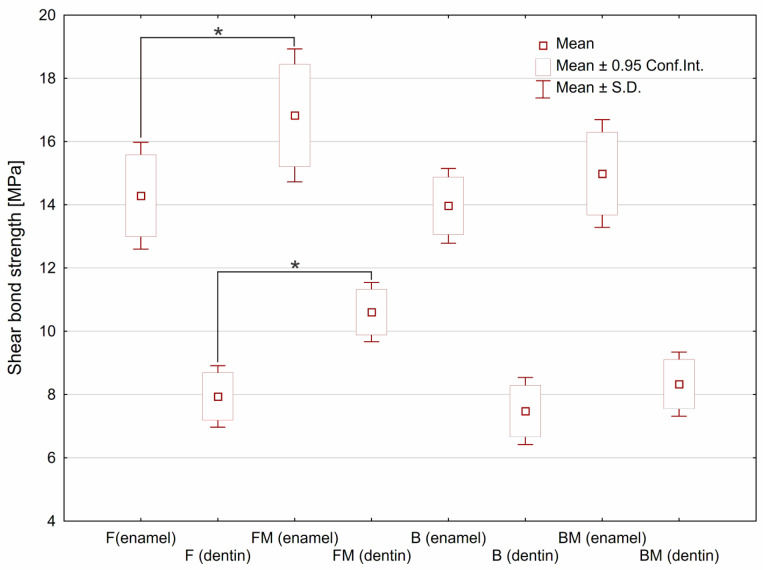
The shear bond strength estimated for tested materials depending on type of the tooth tissue. Asterisks (*) point at statistically significant differences (*p* < 0.05) according to the unpaired *t* test.

Results of SBS tests are presented in Figure 2. Modification of flow type composite (FM sample) resulted in significant increase (*p* < 0.05) of SBS value for both kinds of tooth tissues. The 17% increase in the SBS value for enamel was observed, whereas SBS value for dentine increased by over 33%. In the case of modified packable composite (BM sample), an upward trend was observed however without statistical significance.

The test showed a significant increase in SBS value in the case of flow type dental composite after modification with liquid rubber (FM specimen). It was assumed that observed increase may be related to the reduced viscosity after liquid rubber modification of resin matrix, which was demonstrated in our previous work [7]. An upward trend, however without statistical significance, was observed for modified packable composite (BM). It was noticed that flow type composites showed almost equal SBS values as packable ones. In the case of flowable material a higher SBS value can be explained by better penetration to microirregularities of the substrate surface. Moreover, improved conversion degree obtained after the modification [7] may enhance the connection with the bonding agent.

With a similar chemical composition of the matrix, it can be expected that the SBS value will be comparative to the matrix volume fraction in the composite. It should also be taken into account that in the case of the packable composite, it is possible to form porosity by closing the air bubbles. This fact will facilitate cracking according to Griffith’s theory. However, the SBS values estimated for both packable composites (B and BM) were almost equal to the values obtained for both flow composites (F and FM). The high SBS value obtained for the packable composite may be the result of its higher elastic modulus demonstrated in our previous study [7], leading to more uniform distribution of the stress over the bonded interface. 

### 3.3. Antibiofim Activity 

**Figure 3:**
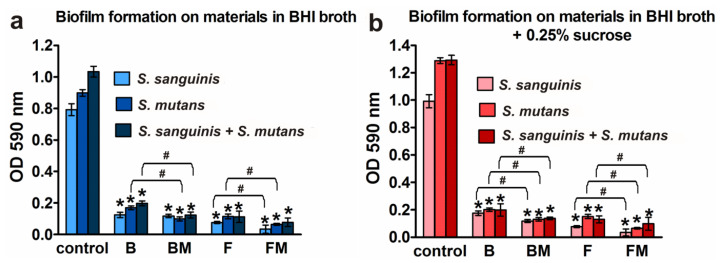
Quantification of the adhered bacteria to the B, BM, F, and FM materials compared to the control surface (polystyrene): (**a**) biofilm formation on materials in BHI broth; (**b**) biofilm formation on materials in BHI broth with 0.25% sucrose; results are shown as mean values ± SD from triplicate experiments; * indicates statistically significant results (*p* < 0.05) compared to the control, # indicates statistically significant results (*p* < 0.05) between unmodified and corresponding modified composite (the unpaired *t* test).

To evaluate the effects of the B, BM, F, FM materials on biofilm formation, composite discs were added to planktonic bacteria (separately *S. mutans*, *S. sanguinis* for mono-species biofilm assay or *S. mutans* with *S. sanguinis* for mixed-species biofilm) and incubated for 48 h. Next, crystal violet staining was used to quantitatively detect biofilm. Figure 3 clearly shows strong reduction of biofilm formation on all tested materials compared to positive control grown on polystyrene. Flow type materials (F, FM) were more resistant (but the results were not statistically significant) to bacterial colonization (regardless of the species and the type of medium) than packable type composites (B, BM) having a higher content of ceramics. Importantly, modified FM material had the most resistant surface to biofilm adhesion among all tested samples. Considering % of positive controls (biofilm formation = 100%), it was less than 7% of mono- or mixed-species biomass grown (both in BHI medium and enriched with sugar) on the FM disc. The inhibition of biofilm formation followed the trend: FM (less than 7%), F disc (less than 12.79%), BM (less than 15.01%), and B (less than 18.9%) against the positive control. 

Figure 3a,b show that addition of 0.25% sucrose to the medium increased (compared to clear BHI broth) the growth of biofilm but mainly in the case of controls grown on polystyrene (increase by 43.3% for *S. mutans*, 25.2% for *S. sanguinis*, and 25.1% for mixed species). Slight increase in biofilm formation in the medium with sucrose was observed also on B material (increase by 1.94% for *S. sanguinis* and by 0.43% for mixed species). Importantly, in the case of both modified dental biomaterials (BM and FM), this extra sugar did not promote biofilm formation.

**Figure 4:**
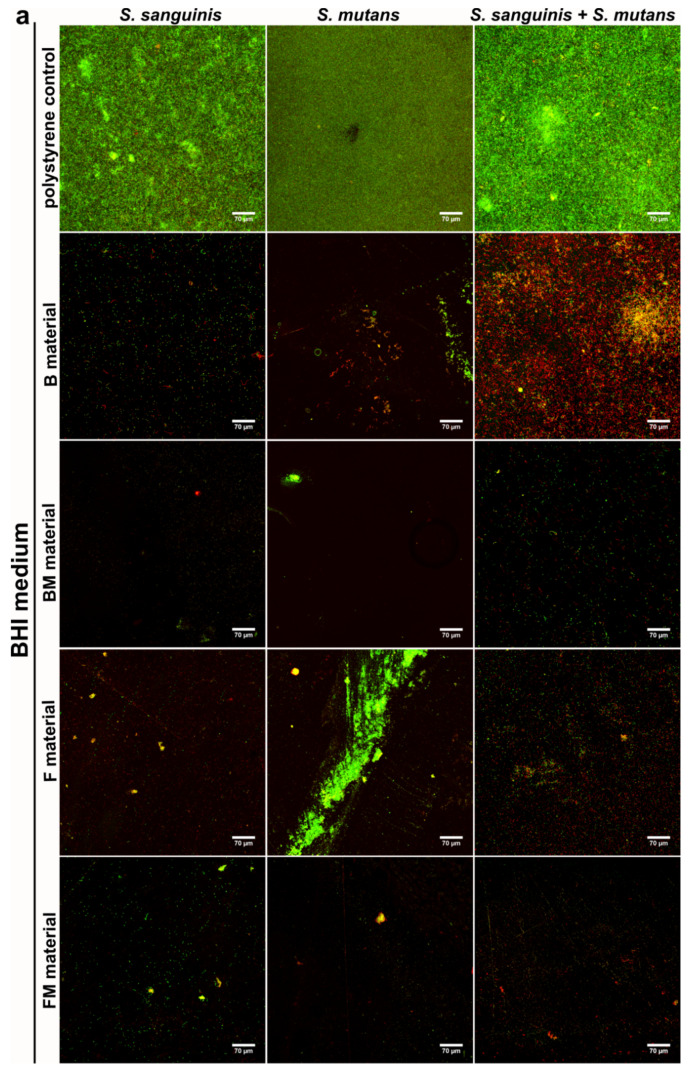

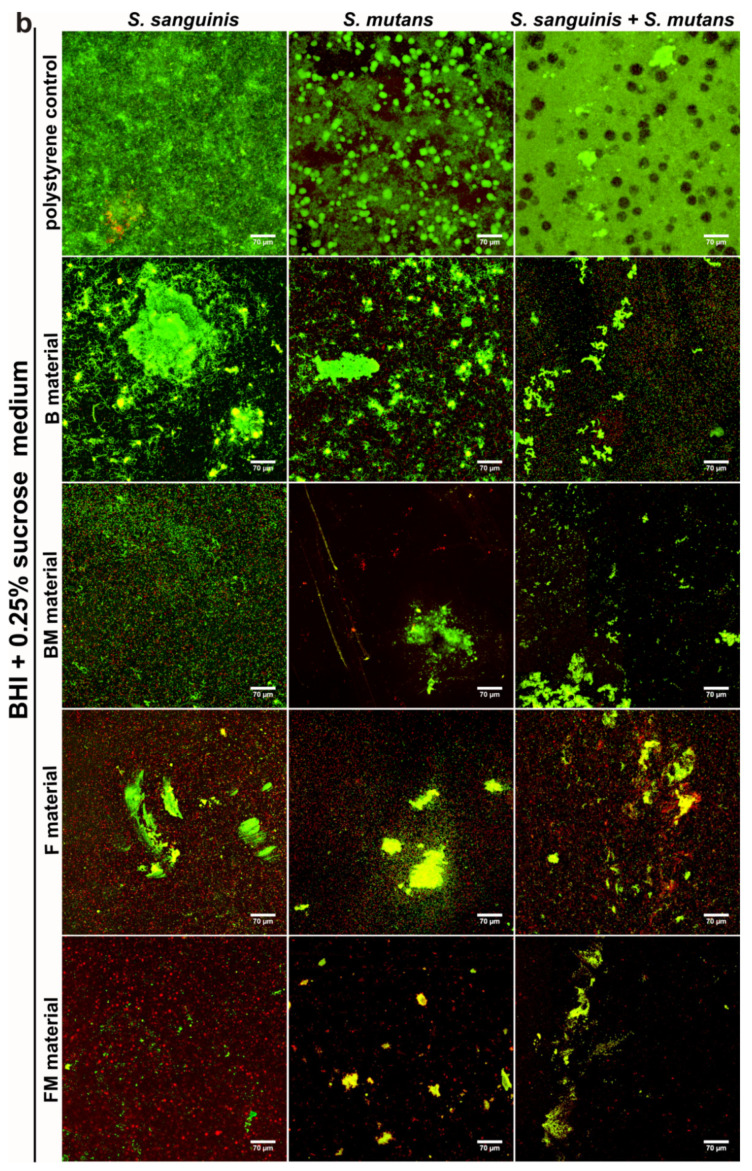
CLSM images of biofilm formed by *S. sanguinis*, *S. mutans*, and mixed species: *S. sanguinis* and *S. mutans* on B, BM, F, FM composite materials in BHI medium (**a**) and in BHI + 0.25% sucrose medium (**b**). Magn. 400×, scale bar = 70 µm (green fluorescence—viable cells, yellow and red fluorescence—dead cells).

Results obtained with the quantitative assay were next confirmed by biofilm determination with the use of CLSM. The CLSM images presented in Figure 4a,b show *S. mutans* and *S. sanguinis* mono- and mixed-species biofilm on the materials surface with viable (stained green) and dead colonies (stained yellow-red). Surface of the disc without biofilm is visible as a non-stained area (no fluorescent signal—black color).

The control group demonstrated viable bacteria after 48 h of incubation in BHI and BHI with sucrose, which formed green fluorescent colonies, proving that bacteria were viable during the period of evaluation. Whereas dead bacteria (red colonies) and some small green colonies (formed by viable bacteria) were observed on the tested composite discs. The images clearly demonstrate weaker adhesion of the bacteria to the modified FM, BM samples as well as to the unmodified F and B discs in comparison to the control (Figure 4a,b). However, the weakest bacteria adhesion was observed on the FM composite that contained liquid rubber and lower content of ceramics compared to packable type composites. Therefore the images confirmed the quantitative tests, where mono- and mixed-species biofilms showed limited growth on FM, F, BM and B disc surfaces in comparison with control surfaces. Importantly, the FM composite had the most resistant surface to bacterial adhesion.

Periodontal diseases and caries are caused by oral microflora changes and are the most common bacterial diseases occurring in the human and animal oral cavity. Almost 80% of bacterial infections of tissue or implants are associated with biofilm formation. Despite various findings concerning the physical and biochemical parameters of the biofilm-forming bacteria and the surface characteristics of the implant, the need for an ideal material still exists. Within these studies the antibiofilm properties of new dental materials were tested against caries bacteria. The effect of various types of material surfaces (B, BM, F, FM) and sucrose additions on biofilm formation were determined.

There are contrary opinions in the available literature concerning biofilm formation ability of main oral cavity bacteria (*S. sanguinis* and *S. mutans*) dependent on the surface wettability of the dental materials. It was observed that *S. sanguinis* bacteria better adhere to the ceramic materials compared to titanium regardless of the surface hydrophilicity [33]. Interestingly, some papers have described better adhesion and biofilm formation by *S. mutans* on hydrophilic surfaces [37] while majority of the authors proved that *S. mutans* bacteria have hydrophobic character of cell membrane and thus better adhere to the hydrophobic materials [32,38]. Moreover, it was observed that surface topography and chemistry are the key factors responsible for bacterial biofilm formation, whereas wettability is the second-rate issue [33,34].

Unfortunately, there is no clear factor enabling prediction of the probability of bacterial adhesion to the surface of dental materials. However, there are many features of materials’ surface affecting the adhesion of bacteria to the implants, e.g., material chemical composition, its specific texture and topography as well as wettability and physicochemical properties of the surface. In fact, multiple factors are working simultaneously affecting bacterial adhesion. According to available literature it is known that surface roughness and character (hydrophilic or hydrophobic) of oral cavity bacteria wall–rather than material wettability–are the major agents responsible for bacterial adhesion [34,39,40]. This is in agreement with our results which demonstrated that samples modified with liquid rubber disc (FM) were the most resistant to bacterial biofilm formation among all tested samples, although the modification reduced the hydrophilic character. Thus, it is possible that modifications of composites influenced also their surface topography providing improved antibacterial protection. Importantly, all investigated samples had hydrophilic character, which according to many authors limits *S. mutans* and *S. sanguinis* bacteria adhesion [33,41]. Since unmodified samples revealed slightly higher hydrophilicity compared to BM and FM discs, it may be inferred that slightly reduced biofilm formation on modified composites most likely resulted from their different topography compared to unmodified materials.

### 3.4. Evaluation of Materials’ Cytotoxicity

**Figure 5:**
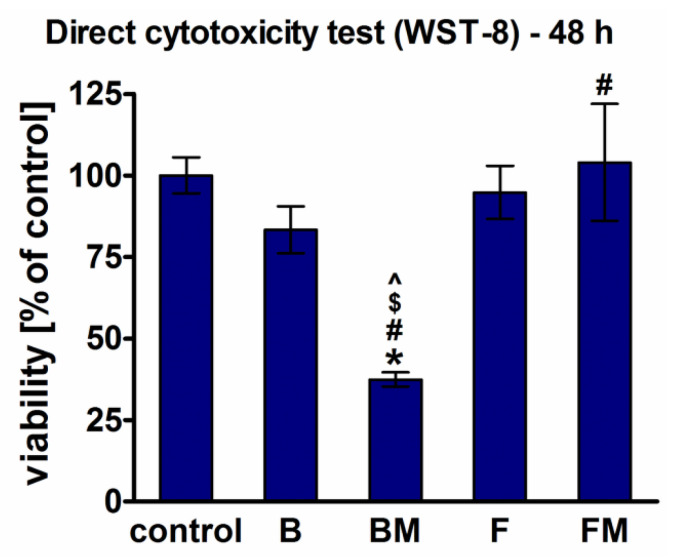
Cytotoxicity of the materials assessed by WST-8 test after direct 48 h contact of BJ fibroblasts with the materials (*significantly different results compared to the negative control of cytotoxicity, #significantly different results compared to the B material, $significantly different results compared to the FM material, ^significantly different results compared to the F material, according to one-way ANOVA test followed by Tukey’s multiple comparison test, *p* < 0.05).

Direct cytotoxicity tests performed after 48-h culture of fibroblasts on the surfaces of the materials demonstrated lack of cytotoxicity only for B (viability = 83%), F (viability = 95%), and FM (viability = 104%) materials, whereas modification of B composite (BM sample) resulted in a significant reduction of cell viability to 37% (Figure 5). 

**Figure 6:**
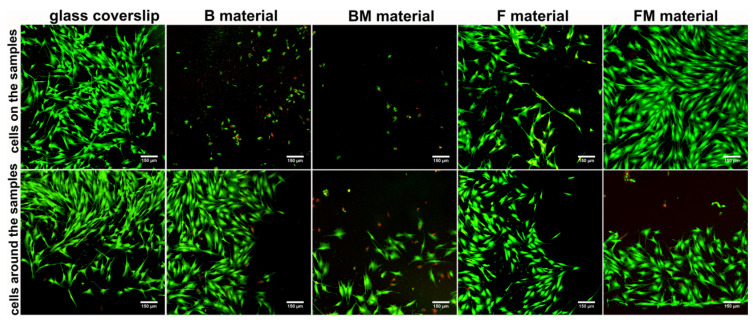
Qualitative evaluation of materials’ cytotoxicity by live/dead double staining of BJ fibroblasts cultured for 48 h on the materials’ surfaces, and grown around the samples (viable cells—green fluorescence, nuclei of dead cells—red fluorescence, apoptotic cells—green and red florescence); magn. 100×, scale bar = 150 µm.

Live/dead staining of fibroblasts cultured directly on the materials showed that B and BM surfaces were not favorable to cell adhesion and growth (Figure 6). Only apoptotic (green and red fluorescence) and dead cells (red fluorescence) were found on the surfaces of these materials. F sample allowed attachment of small amounts of cells, whereas FM material was very supportive to cell adhesion and survival, which is consistent with WST-8 direct cytotoxicity results (Figure 5). Importantly, there was also great number of apoptotic and dead cells around the BM sample, proving cytotoxicity of this material. Cells grown around B, F, and FM materials were viable and had typical fibroblastic morphology. Thus, in the case of B and F samples slight reduction in cell viability (to 83% and 95%, respectively) observed in quantitative direct contact test (WST-8) primarily resulted from hindered fibroblast adhesion to the surfaces of these materials. 

Within these studies cytotoxicity was assessed in direct contact with the tested composites. WST-8 quantitative assay showed that fibroblasts revealed the highest viability on the flow type samples (FM and F) which showed also the highest ability to inhibit biofilm formation, proving their potential in biomedical applications. Moreover, CLSM observation demonstrated that FM surface was the most supportive to cell adhesion and growth among all test samples. 

It is well known that hydrophilic surfaces of the biomaterials, revealing low water CA, promote the adsorption of cell adhesion proteins (e.g., fibronectin, laminin), allowing for more effective eukaryotic cell adhesion [21,35,42]. Interestingly, wettability tests showed higher hydrophilicity of the B and F samples compared to modified BM and FM composites. However, the B and BM samples did not allow for attachment of any BJ cell and F composite hindered cell adhesion. Therefore, surface hydrophilicity was not the key factor responsible for biocompatibility of fabricated composites. It should be noted that also other features of materials’ surface influence cell adhesion, e.g., surface chemistry, charge or roughness, especially at nanometric scale [43,44]. All composites showed flat and rather smooth surfaces, thus sensitive analysis such as atomic force microscopy (AFM) should be performed to evaluate differences in nanoroughness between the samples. Nevertheless, it may be assumed that slightly lower amount of BJ fibroblasts attached to the F sample compared to modified FM composite most likely resulted from its smoother surface. The only difference between B and BM composites was the presence of additional component in BM material (5% of a liquid methacrylate-terminated polybutadiene), which improved biocompatibility of F sample. Thus, the reason of cytotoxic effect of only BM sample is hard to be explained without detailed analysis of surface chemical composition with the use of ATR-FTIR or XPS, which are planned to be performed in our future studies.

## 4. Conclusions

Results of the studies described herein revealed that the modification of packable type resin-based material (B) with liquid rubber reduced its hydrophilicity, improved its antibiofilm activity against *Steptococcus mutans* and *Streptococcus sanguinis*, but made the resultant BM material cytotoxic against eukaryotic cells. Interestingly, the introduction of the same modification to a flow type resin-based material (F)—which had lower content of the ceramic filler compared to the packable type–allowed us to obtain a novel dental material (FM) with higher adhesion to the tooth tissues, reduced hydrophilicity, significantly improved ability to inhibit bacterial biofilm formation and nontoxicity against human fibroblasts. Based on the obtained results it may be concluded that the fabricated FM composite can be a candidate to be used as restorative material since it exhibits nontoxicity and possesses antibiofilm properties, which are the best among all tested composites.

## Data Availability

The raw/processed data required to reproduce these findings can be obtained from the corresponding authors (k.palka@pollub.pl and agata.przekora@umlub.pl) upon reasonable request.
